# Glioblastoma multiforme with vertebral metastases: A case report

**DOI:** 10.1111/cns.13785

**Published:** 2021-12-30

**Authors:** Hongyu Liu, Chuanbiao Chen, Fangye Li, Yangrui Zheng, Jialin Liu, Xinguang Yu, Ling Chen

**Affiliations:** ^1^ Department of Neurosurgery Hainan Hospital of Chinese PLA General Hospital Sanya China; ^2^ Department of Neurosurgery First Medical Center of Chinese PLA General Hospital Beijing China

## CONFLICT OF INTEREST

The authors declare no conflicts of interest.

Dear Editor:

Glioblastoma multiforme (GBM) is the most aggressive and lethal form of brain cancer.^1^ The currently recommended treatment guideline consists of maximal surgical resection with adjuvant radiotherapy and temozolomide (TMZ) chemotherapy. However, GBM patients still have a poor prognosis with a 5‐year survival rate less than 10%.^1^ GBM‐associated vertebral metastases are extremely rare.^2^ Here, we report a rare case of thoracic vertebral metastases in the absence of intracranial recurrence.

**FIGURE 1:**
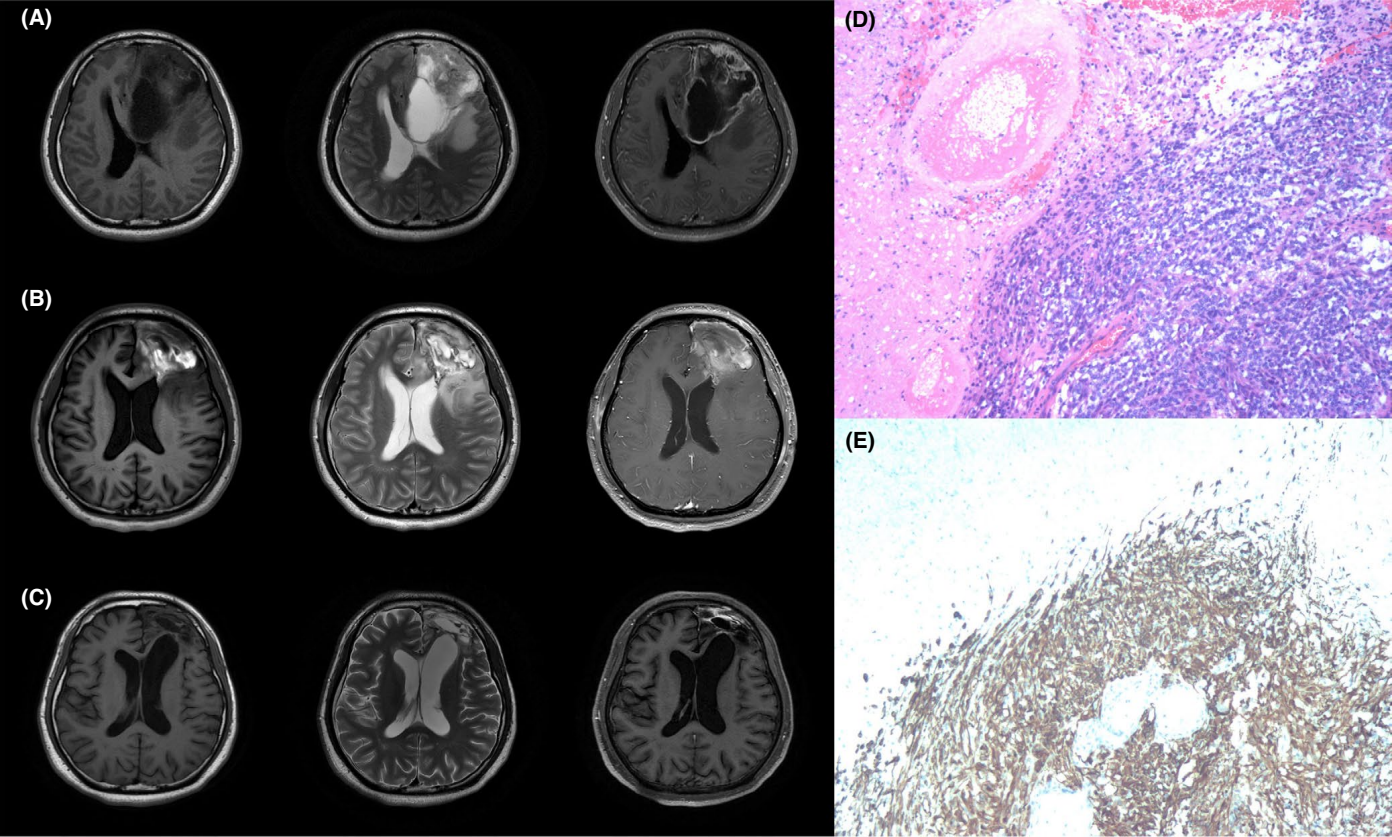
MRI images and pathological results of the brain lesion. (A) Preoperative brain MRI images on June 1, 2020. (B) Postoperative brain MRI images on June 19, 2020. (C) Postoperative brain MRI images on April 2, 2021. (D) Histopathological examination of the primary brain lesion. (E) Immunohistochemical staining of the primary brain lesion with GFAP

On May 28, 2020, a 25‐year‐old man with an average medical history was admitted to our hospital complaining of intermittent dizziness and headache for 2 months, in addition to a progressively decreased left vision for 3 months. Neurological examination revealed cognitive decline and left vision loss (only light perception). Preoperative brain magnetic resonance imaging (MRI) showed an irregular mass measuring 87 mm × 58 mm × 60 mm in the left frontotemporal lobe (Figure 1A). The tumor presented a hypointense signal on the T1‐weighted sequence and a hyperintense signal on the T2‐weighted sequence. The left ventricle was compressed significantly, and the lesion was enhanced heterogeneously on the T1 contrast sequence with surrounding edema and central necrosis (Figure 1A). Surgical resection was performed without complications under neuronavigation assistance via the left frontotemporal approach on June 7, 2020. Following successful surgery, the headache and dizziness disappeared, and the left vision was improved slightly, with an uncorrected visual acuity of counting fingers 30 cm.

The pathological diagnosis was high‐grade glioma with necrosis, and the partial area was GBM (WHO IV) (Figure 1D). Immunohistochemistry (IHC) showed a strong positive signal for glial fibrillary acidic protein (GFAP) (Figure 1E). Isocitrate dehydrogenase 1 (IDH‐1) was wild‐type, and the methylation of O6‐methylguanine‐DNA‐methyltransferase (MGMT) promoter was positive. On June 19, 2020, postoperative re‐examination of the brain MRI showed that the tumor had been completely removed, and there was less chronic hematoma in the operative region (Figure 1B). The patient received adjuvant radiation therapy with a total dose of 60 Gy (2 Gy given 5 days per week for 6 weeks) and chemotherapy with TMZ (75 mg/m^2^ per day for 6 weeks) from June 29, 2020, to July 31, 2020. Brain MRI performed before discharge showed no recurrence. Periodic TMZ chemotherapy was subsequently performed (150 mg/m^2^ for 5/28 days).

**FIGURE 2:**
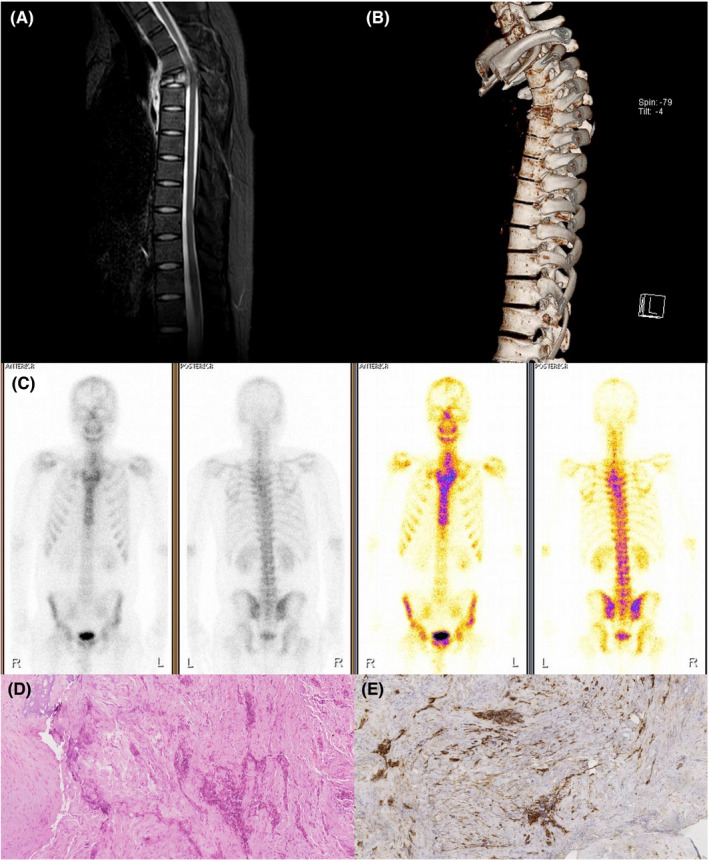
Imaging manifestations and pathological results of the T4 vertebral lesion. (A) MRI of the thoracic vertebrae. (B) 3D reconstruction of the thoracic vertebrae. (C) The PET/CT results. (D) Histopathological examination of the vertebral lesion. (E) Immunohistochemical staining of the vertebral lesion with GFAP

During the 7th cycle of TMZ chemotherapy, the patient complained of a local pain in the lower back. MRI‐mediated assessment of the thoracic vertebrae on April 6, 2021, revealed a pathological fracture located in the T4 vertebra (Figure 2A), which was also confirmed by 3‐dimensional (3D) reconstruction on computed tomography (CT) (Figure 2B). In addition, positron emission tomography/computed tomography (PET/CT) showed an abnormal signal in the thoracic vertebrae (Figure 2C). However, re‐examination of the brain MRI showed no tumor recurrence (Figure 1C). Members of a multidisciplinary consultation board at our hospital diagnosed a metastasized form of GBM.

Subsequently, thoracic lesion resection was performed at the People's Hospital of Peking University on May 19, 2021. The postoperative pathology revealed numerous tumor cells staining positive for GFAP that were patchily distributed in the bone trabecula, which confirmed the bone metastasis from brain GBM (Figure 2D,E). The patient experienced symptom alleviation after the surgery, and the 8th TMZ chemotherapy cycle was subsequently resumed on July 7, 2021.

Extracranial GBM metastases are rare, with incidences reported from 0.4 to 2%.^3^ Possible explanations for the lack of metastatic dissemination in previous studies are a short survival time, the presence of the blood‐brain barrier, and, previously, the absence of lymphatics in the brain.^3^ The discovery of central nervous system functional lymphatic vessels overturned our comprehension that there was no classical lymphatic drainage system in the brain, which provides a theoretical basis for glioma cells to migrate to deep cervical lymph nodes.^4^ Recent studies also revealed the existence of a glymphatic system. This fluid clearance pathway drains to meningeal and cervical lymphatic drainage vessels, which can play an essential role in GBM metastasis.^5^, ^6^, ^7^ However, the highly invasive growth and poor prognosis lead to too short overall survival time to form metastases.^8^ Furthermore, craniotomy and other invasive procedures, such as biopsy, could break the blood‐brain barrier, creating conditions for glioma cells to migrating into the systemic circulation.^9^ Most metastases cases reported in previous studies involved primary tumor resection before the formation of extracranial metastases.^8^, ^9^

Recent studies have demonstrated that genetic alterations are related to GBM‐derived bone metastases, such as mutation of in BRCA1, ARID1A, and C8A‐R30W, or overexpression of IGFBP2.^10^, ^11^, ^12^ We identified several GBM metastasis‐related genes described in previous studies. The LASSO Cox regression model determined six genes (IGFBP2, GNS, LBH, SCARA3, EGFR, and MLH1) with the best prognostic value. In addition, bioinformatics, and Kaplan‐Meier and ROC curve analyses demonstrated that the risk signature associated with these six genes could be an essential reference to predict the prognosis of GBM patients (Figure S1).

An additional aspect worth considering is that GBM cells need to evade peripheral immunosurveillance to form extracranial metastases. A recent case report described a patient primarily diagnosed with IDH‐wild‐type GBM and treated with immune checkpoint inhibitors. The patient presented with multiple vertebral metastases, and complete remission of intracranial tumors was achieved. Detection of the increased exhaustion markers KLRG1 and CD57 implied that the peripheral immune system was functionally impaired. This study established the potential mechanisms of immune escape in GBM and metastatic tumor evolution.^13^

Goodwin et al. reported that the most common site of GBM‐derived vertebral metastases was the thoracic vertebrae (32%), and 28.6% of these metastases were located at more than one vertebral level. Less common metastasis sites are the lumbar vertebrae (17.9%) and cervical vertebrae (10.7%).^2^ The mean durations from diagnosis of GBM to detection of extracranial metastases and from vertebral metastasis to mortality were 26.4 months and 10 months, respectively.^2^

Histopathological examination and immunohistochemical staining provide essential evidence for a precise diagnosis. Microscopically, compressed tumor cells are always mixed with bone trabeculae and proliferative fibrous tissue. GFAP is a specific and sensitive marker for the pathological diagnosis of vertebral metastasis.^2^ Bligin et al. retrospectively analyzed 214 GBM patients post‐surgery. Among them, the incidence of spinal metastasis was 1.91%, and all the patients with metastasis had wild‐type IDH with significantly increased Ki‐67 index, which indicated that spinal screening should be performed in GBM patients with high Ki‐67 index and wild‐type IDH.^14^

Surgical treatment is beneficial in patients with vertebral metastases. The primary purpose of surgery is to remove the lesion, to prolong the survival time, and to relieve the compression of the spinal cord to alleviate excruciating pain, significantly improving the quality of life. Therefore, it is vital to enquire about patient preferences and weigh surgical risks and benefits, especially in patients with advanced, recurrent GBM. Radiation therapy and chemotherapy supplemented with analgesics are also effective treatments for pain relief and delayed progression.

In conclusion, we report a rare case of GBM with vertebral metastases in the absence of intracranial recurrence. Advances in therapeutic methods have prolonged the overall survival of GBM patients, and the incidence of vertebral metastases may increase. Although vertebral metastases are not the leading cause of death and cannot significantly affect the prognosis of GBM patients, they should be taken into consideration during the treatment evaluation. Early detection and timely treatment are crucial for improvement of the overall prognosis. In conclusion, the epidemiology and pathogenesis of GBM‐associated extracranial and, in particular, vertebral metastasis deserves further investigation.

## Supporting information

Fig S1

## Data Availability

All data associated with this study are present in the paper or the Supplementary Materials.
