# Supplementary figures and images for: Glioblastoma multiforme with vertebral metastases: A case report
Source: CNS Neurosci Ther. 2021 Dec 30;28(2):310–3. doi: 10.1111/cns.13785 (PMC8739038; doi:10.1111/cns.13785)

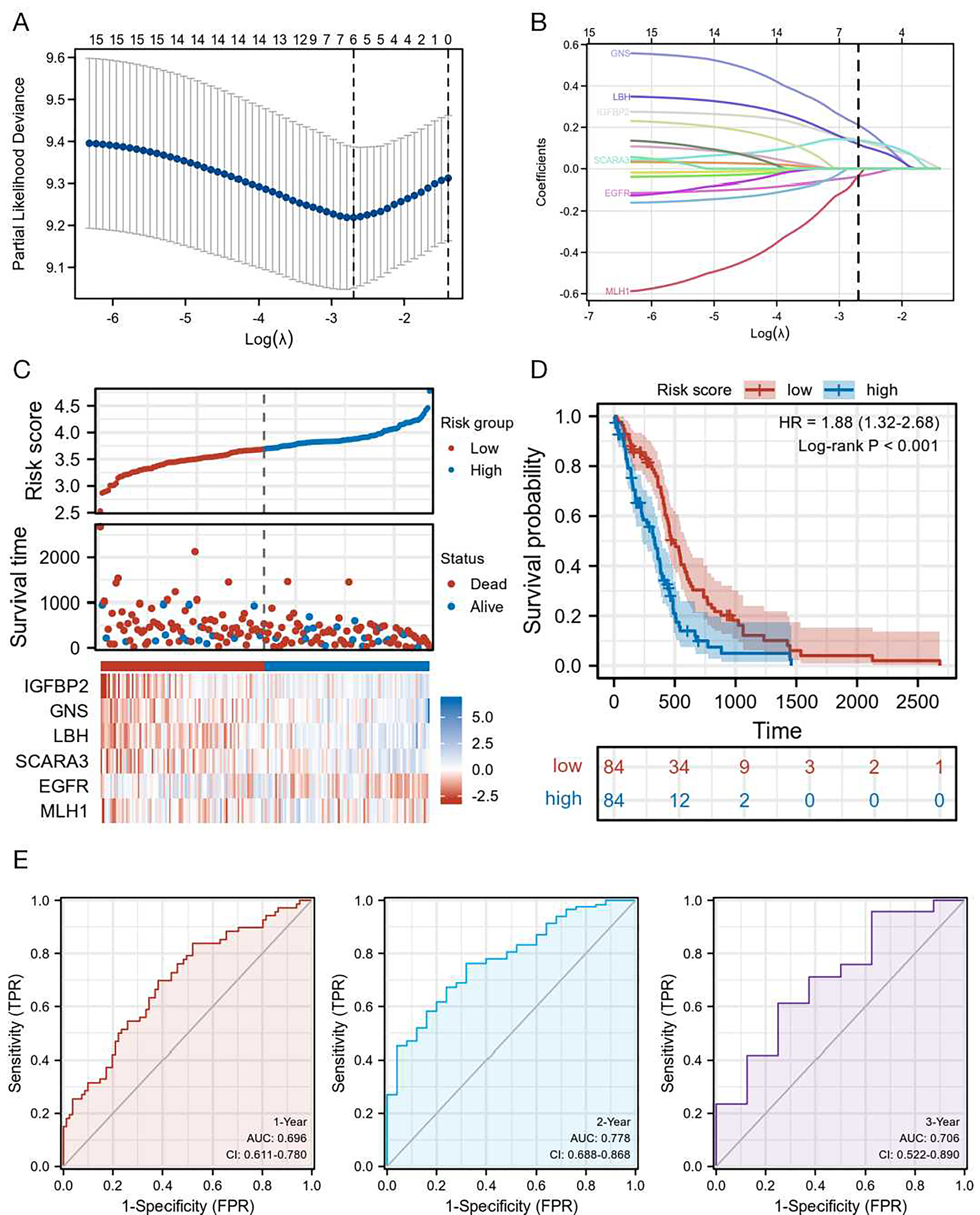

Supplement: Supplementary file 1 — Fig S1 [file CNS-28-310-s001.jpeg]
